# MANDATORY MEDICAID MANAGED LONG-TERM SERVICES AND SUPPORTS IN PENNSYLVANIA: EVALUATING POLICY CHANGE

**DOI:** 10.1093/geroni/igz038.849

**Published:** 2019-11-08

**Authors:** Howard Degenholtz

**Affiliations:** University of Pittsburgh, Pittsburgh, Pennsylvania, United States

## Abstract

The Commonwealth of Pennsylvania is implementing a mandatory Medicaid managed Long-Term Services and Supports (LTSS) program that covers people age 21 and older who are fully eligibly for both Medicare and Medicaid, living in a nursing facility paid for by Medicaid, or in an aged or physical disability home and community based services (HCBS) waiver. The overall program goals are to: Enhance Opportunities for Community Living; Improve Service Coordination; Enhance Quality and Accountability; Advance Program and Innovation; and Increase Efficiency. The program will be administered by 3 managed care organizations (MCOs) that are obligated to coordinate with Medicare Advantage and D-SNP plans. This major policy change affects the traditional roles and responsibilities of the aging network by shifting the locus of control to insurance companies. This presentation will describe the policy change, the implications for the aging network, and the multi-method evaluation designed to assess the implementation and outcomes.

